# Supplementary material for: Differentiation of human induced pluripotent stem cells into cortical neural stem cells
Source: Front Cell Dev Biol. 2023 Jan 5;10:1023340. doi: 10.3389/fcell.2022.1023340 (PMC9849742; doi:10.3389/fcell.2022.1023340)
Supplement: Supplementary file 1 [file DataSheet2.PDF]

Generation of knockout iPSCs using CRISPR/Cas9

In order to create knockout iPSC lines, guide RNAs (gRNAs) are selected which target a conserved exon, preferably early in the protein coding sequence of a gene. For each gene target, two gRNAs are designed to account for the often unpredictable editing efficiency at different loci. The synthetic gRNA along with the Cas9 protein are delivered into the cells as a pre-complexed ribonucleoprotein via electroporation. The addition of a short single-stranded oligodeoxynucleotide of non-complementary sequence is also added to improve delivery. After a period of recovery, the cells are subcloned and up to 192 colonies picked and submitted for Next Generation Sequencing (NGS). A T7 endonuclease assay is used to assess the efficiency and inform the number of colonies needed. Clones are screened for the presence of frameshift-causing indels (insertions or deletions) and then expanded for banking. For each gene target we aim to expand both heterozygous (HT) and homozygous (HM) knockouts, as well as wild-type clones to act as controls. The wild-type clones have gone through the same treatment as the heterozygous and homozygous cells, but during the screening process are identified as non-edited. This importantly means the wildtypes should be equivalent in every other way except for the knockout mutation. After banking, the cells are sent for a second round of NGS to confirm the identity of the mutation.

The cell editing and banking work was performed by the Gene Editing facility at the Wellcome Sanger Institute.
